# Evaluation of bi-atrial dynamic electrophysiological properties in atrial septal defect patients

**DOI:** 10.3389/fphys.2025.1705582

**Published:** 2026-02-13

**Authors:** Louisa O’Neill, Keeran Vickneson, Ali Gharaviri, Iain Sim, Daniel O’Hare, John Whitaker, Rahul Mukherjee, Steven Niederer, Una Buckley, Matthew Wright, Matthew Jones, Eric Rosenthal, Alessandra Frigiola, Vivienne Ezzat, Steven E. Williams, Mark D. O’Neill

**Affiliations:** 1 School of Biomedical Engineering and Imaging Sciences, King’s College London, London, United Kingdom; 2 Department of Cardiology Guy’s and St Thomas’ NHS Foundation Trust, London, United Kingdom; 3 Institute for Neuroscience and Cardiovascular Research, The University of Edinburgh, Edinburgh, United Kingdom; 4 Barts Heart Centre, London, United Kingdom

**Keywords:** atrial septal defect, right atrium, voltage mapping, atrial refractory period, conduction velocity, atrial arrhythmia

## Abstract

**Background:**

Atrial septal defects (ASD) are associated with an increased incidence of atrial arrhythmias, but their electrophysiological consequences are poorly defined. We hypothesised that conduction and repolarisation would be preferentially altered in the right atrium of ASD patients.

**Objective:**

To quantify atrial conduction and repolarisation in ASD patients and determine the impact of structural remodelling on restitution properties.

**Methods:**

Patients with an ASD (n = 22) underwent bi-atrial electroanatomic mapping and quantification of effective refractory periods, longitudinal and transverse local conduction. The control group comprised 24 patients without an ASD undergoing ablation for paroxysmal AF.

**Results:**

Bipolar voltage was significantly lower in ASD patients (right atrium: 1.53 ± 0.46 mV versus 1.98 ± 0.59 mV, P = 0.017; left atrium: 1.71 ± 0.36 mV versus 2.06 ± 0.63 mV, P = 0.039). There was no significant difference in global conduction velocity in either atrium between ASD and control patients. Effective refractory periods at 600 ms were not significantly different between patient groups (right atrium: 247 ± 34.7 ms versus 224 ± 36.5 ms, P = 0.071; left atrium: 244 ± 23.9 ms versus 232 ± 40.4 ms, P = 0.29). However, both conduction and repolarisation demonstrated greater rate adaptation in ASD patients in both atria.

**Conclusion:**

Right atrial remodelling, characterised by atrial dilatation and increased low voltage, is present in ASD patients. During fixed rate pacing, conduction and repolarisation properties are similar between ASD and AF patients. However, the restitution properties of both conduction and repolarisation are more pronounced in ASD than AF patients.

## Introduction

Atrial fibrillation is more prevalent in patients with an uncorrected secundum atrial septal defect (ASD) compared to the general population (Chubb et al., 2014). The incidence of atrial arrhythmias rises with age from ∼10% in those under 40 to >20% in those over 40 (Vecht et al., 2010). Despite this increased risk, little is known about the electrophysiological basis of atrial arrhythmias in adults with an uncorrected ASD. In a total of only 24 patients, two prior studies have reported reduced left atrial (LA) voltage, prolonged bi-atrial conduction times and unchanged or prolonged bi-atrial refractory periods compared to patients without atrial fibrillation (AF) (Roberts-Thomson et al., 2009; Morton et al., 2003). These studies used traditional electrophysiology measurements using multipolar catheters to assess conduction and repolarisation. Remarkably, there have been no electroanatomic mapping studies comparing the electrophysiological properties of both atria in patients with and without ASDs. The only left atrial electroanatomic mapping study pre-dates contemporary high-density mapping, with a mean of <100 mapping points per case (Roberts-Thomson et al., 2009).

In contrast, the electrophysiological basis of AF in patients without congenital heart disease has been extensively studied (Sim et al., 2019). Dynamic atrial electrophysiological properties have been associated with both AF vulnerability and arrhythmia recurrence following catheter ablation. Key amongst these properties are conduction velocity (CV), action potential duration and their restitution properties (Narayan et al., 2008). Atrial structural remodelling has also been extensively studied in patients without congenital heart disease, with correlation demonstrated between regional electrophysiological and structural abnormalities including atrial fibrosis (Teh et al., 2012). Using cardiac MRI we have reported increased right atrial fibrosis in ASD patients, compared to non-congenital heart disease patients with AF, (O'Neill et al., 2022a) consistent with prolonged right atrial (RA) stretch. In our previous study, RA fibrosis was associated with the presence of atrial arrhythmias, suggesting that electrophysiological changes may also predominate in the RA of patients with an ASD.

We therefore hypothesised that dynamic conduction and repolarisation properties would be preferentially altered in the RA of ASD patients. We aimed to quantify CV, effective refractory periods and their restitution properties in both the left and right atrium of ASD patients over the age of 40 years, compared to a control group of non-congenital heart disease patients undergoing catheter ablation for AF. Furthermore, we used electroanatomic mapping to determine the impact of atrial structural remodelling on atrial conduction in ASD patients.

## Methods

This prospective cohort study conformed to the principles of the Declaration of Helsinki. Ethical approval was granted by the Health Research Authority and the London and Surrey Borders Research Ethics Committee (17/LO/1218). Informed written consent was obtained from all participants. Consecutive adult patients >40 years with an uncorrected secundum ASD undergoing percutaneous closure underwent atrial electrophysiology assessment (ASD group). Comparison was made to a consecutive group of paroxysmal AF patients undergoing first-time catheter ablation (Control group). Patients with co-existing structural heart disease were excluded.

### Procedural setup

All procedures were performed under general anaesthesia. Following vascular access, a 6Fr decapolar catheter (Inquriy, Abbott) was positioned in the coronary sinus. An 8Fr multielectrode catheter (Pentaray, Biosense Webster, Diamond Bar, California) was used to map both atria in all patients, via the ASD in the ASD group and via transseptal puncture in the control group. Heparin was administered to achieve a target Activated Clotting Time of 350 s.

### Electroanatomic mapping

Bi-atrial bipolar voltage and local activation time mapping during proximal coronary sinus pacing (cycle length 500 ms) was performed using the Pentaray catheter and the Carto3 electroanatomic mapping system (Biosense Webster, Diamond Bar, California). Catheter stability (4 mm/s), local activation time (LAT) stability (<4 ms between neighbouring points) and tissue proximity filters ensured uniform collection of points. Each electrogram was reviewed manually and assessed for inclusion based on previously described criteria (Maille et al., 2018). The thoracic veins and valve annuli reconstruction were removed using the mapping system tools. Quantitative analysis was performed using OpenEP (v1.0.03, https://openep.io) (Williams et al., 2021) to calculate mean chamber voltage, low voltage area <0.5 mV, atrial volume and atrial surface area. Low voltage area was indexed to the LA (or RA) surface area.

### Conduction velocity assessment

For quantification of global CV, radial basis interpolation using OpenEP was performed as previously described (Whitaker et al., 2023). Regions of wave collision were excluded using the divergence of the CV vector field.

**FIGURE 1:**
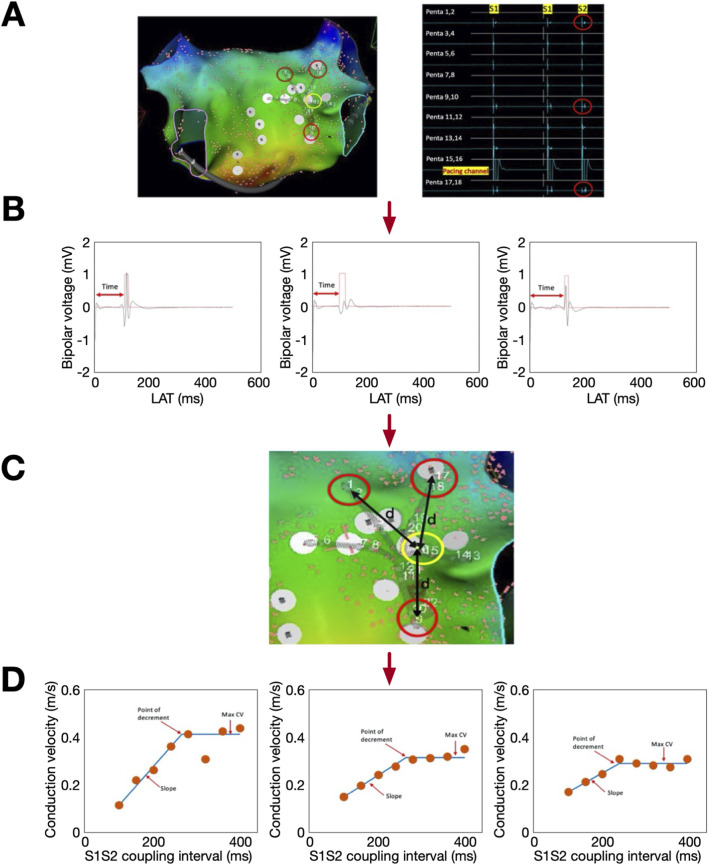
CV and CV restitution calculation. **(A)** Pentaray position with pacing channel highlighted yellow and recording channels highlighted red. Corresponding electrograms from last two beats of the drive train + extra-stimulus are shown. Time from pacing stimulus to local electrograms on the extra-stimulus beat **(B)** and distance between the pacing and recording electrodes **(C)** were measured using OpenEP. **(D)** Example CV restitution curves.

For quantification of local CV, CV restitution and conduction anisotropy, the Pentaray catheter was positioned in either the posterior left atrium or posterior right atrium, with splines splayed. An S1S2 pacing protocol was delivered from a central bipole with S1 = 600 ms × 8 beats and S2 decreasing from 400 ms to loss of 1:1 atrial capture. Analysis was performed as previously described (Figure 1) (Gharaviri et al., 2022) For each Pentaray bipole pair, resulting electrograms were exported from the recording system and analysed in OpenEP to automatically identify the time from pacing spike to earliest activation defined using a non-linear energy operator. Using the location points assigned to each electrode in the electroanatomic mapping system, the distance between pacing and recording electrodes was calculated. The resulting conduction velocities were plotted against S1S2 intervals to generate CV restitution curves. From these, the maximum CV and the slope of the steepest part of the restitution curve were obtained.

For each pacing site, analysis was repeated for the Pentaray spline recording the highest local CV (to measure longitudinal conduction) and the Pentaray spline recording the lowest local CV (to measure transverse conduction) (Linnenbank et al., 2014).

### Repolarisation assessment

Bi-atrial effective refractory periods were measured from a proximal electrode of the Pentaray catheter at the posterior LA and lateral RA at 3 basic cycle lengths of 600 ms, 450 ms and 300 ms. Pacing output was set to twice threshold. If 1:1 atrial capture was not obtained an alternative electrode was chosen. Atrial ERP was measured using an 8 beat drive train with a single extra-stimulus decrementing in 20 ms from 400 ms to loss of atrial capture. Once loss of capture occurred, the extra-stimulus was increased by 10 ms to define the ERP within a 10 ms interval. Atrial ERP was defined as the longest coupling interval failing to capture the atria.

### Statistical analysis

Data analysis was performed using SPSS statistics (IBM, Version 24) and Prism (GraphPad Software, Version 7). Normally distributed continuous variables were expressed as mean ± standard deviation. Non-normally distributed or non-continuous, ordinal data are presented as median (interquartile range). Categorical data are presented as percentages. Comparison of means between groups was performed using independent samples T-test for normally distributed data and Mann-Whitney U test for non-uniformly distributed data. To determine whether the extent of atrial fibrosis defined by voltage mapping independently predicted atrial CV, logistic regression was performed controlling for age and comorbidities. Throughout, P < 0.05 was considered statistically significant.

## Results

### Patient characteristics

Atrial electrophysiological assessment was performed in 22 ASD patients (7 male) and 23 control patients (17 male) (Table 1). Mean age was significantly lower in the ASD group than in the control group (P = 0.027). Within the ASD group, 6 patients had a history of documented atrial arrhythmia consisting of three with paroxysmal AF, two with persistent AF and one with atrial flutter. Mean Qp: Qs in the ASD group was 2.5 ± 0.8. Right atrial area and volume were significantly greater in the ASD group than the control group (Area: 180 ± 39 cm^2^ vs. 143 ± 22 cm^2^, P = 0.0004; Volume: 182 ± 65 mL vs. 84 ± 84 mL, P < 0.0001). There were no significant differences in left atrial area and volume in the ASD group compared to the control group (Area: 143 ± 22 cm^2^ vs. 134 ± 20 cm^2^, P = 0.83; Volume: 73 ± 26 mL vs. 83 ± 17 mL, P = 0.15).

**TABLE 1:** Study population demographic data.

	ASD (n = 22)	Control (n = 23)	P value
Age (years)	50.6 ± 13.1	59.1 ± 11.8	0.027
Male sex (n, %)	7 (30.4)	17 (73.9)	0.006
Hypertension (n, %)	5 (21.7)	7 (30.4)	0.559
Diabetes (n, %)	1 (4.3)	1 (4.3)	0.975
Stroke/TIA (n, %)	0 (0)	2 (8.7)	0.157
CCF (n, %)	0	0	-
CAD (n, %)	0 (0)	2 (8.7)	0.157
CHA_2_DS_2_VaSc (mean)	1.1 ± 06	1.2 ± 1.1	0.849
RA point density (points/cm^2^)	9.3 ± 3.6	8.2 ± 5.1	0.136
LA point density (points/cm^2^)	14.0 ± 4.5	14.0 ± 6.2	0.677

ASD, atrial septal defect; TIA, transient ischaemic attack; CCF, congestive cardiac failure; CAD, coronary artery disease.

### Electroanatomic mapping

**FIGURE 2:**
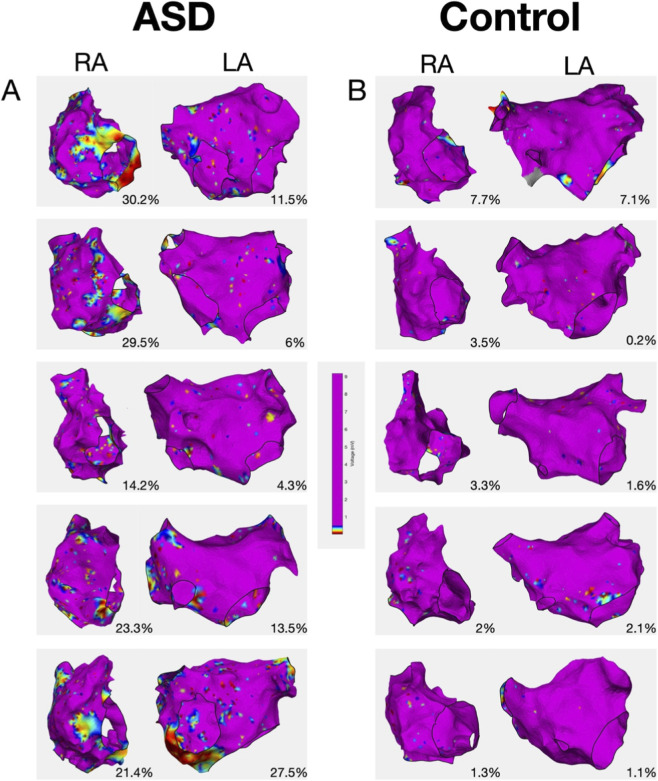
Representative bipolar voltage maps from **(A)** ASD group and **(B)** control group.

**FIGURE 3:**
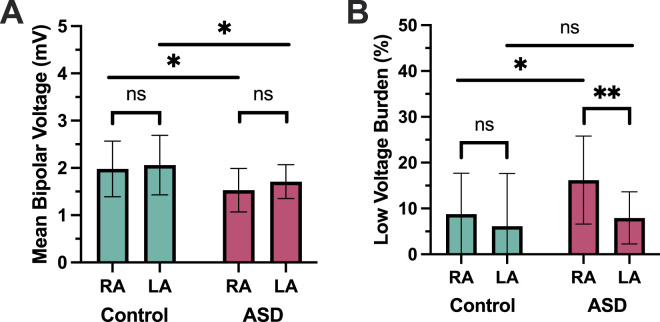
Voltage mapping. **(A)** Mean bipolar voltage. **(B)** Low voltage area. ASD = atrial septal defect; RA = right atrium; LA = left atrium.

During electroanatomic mapping, 1617 ± 541 RA and 1790 ± 355 LA points were collected in ASD patients whilst 1055 ± 551 RA and 1813 ± 700 LA points were collected in AF patients (Figure 2). There was no significant difference in the point density between ASD and AF patients in either the right or left atria (Table 1). In ASD patients, the mean RA voltage was significantly lower (RA (ASD) 1.53 ± 0.46 mV versus RA (control) 1.98 ± 0.59 mV, P = 0.017) (Figure 3A) and the proportion of RA area with a voltage <0.5 mV was greater (16.2% ± 9.6% versus 8.78% ± 8.9%, P = 0.012) compared with the control group (Figure 3B). Although the mean LA voltage was also significantly lower in ASD patients, the magnitude of that difference was lower than for the RA (LA (ASD) 1.71 ± 0.36 mV versus LA (control) 2.06 ± 0.63 mV, P = 0.039). The extent of low voltage in the LA (<0.5 mV) did not differ between ASD and AF patients (7.95% ± 5.7% versus 6.14% ± 11.51%, P = 0.52).

Within the ASD group, the low voltage area was significantly greater in the RA than in the LA (P < 0.01); right and left atrial voltages did not differ significantly in the control group (P = 0.42).

### Global and local conduction velocity

Between the ASD and control groups, mean atrial CV did not differ in either the right (0.60 ± 0.07 m/s vs. 0.56 ± 0.12 m/s, P = 0.22) or left (0.65 ± 0.06 m/s vs. 0.66 ± 0.14 m/s, P = 0.66) atria. Within both the ASD and control groups, RA CV was significantly lower than LA CV (ASD P = 0.029; control P = 0.023).

For the assessment of local CV, there were contrasting observations for longitudinal and transverse conduction, as follows.

**FIGURE 4:**
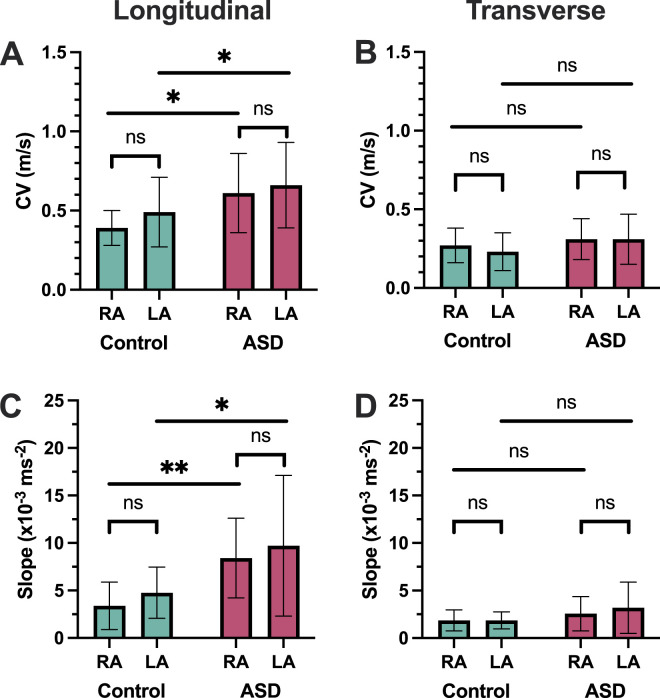
CV. **(A,B)** Longitudinal and transverse local CV. **(C,D)** Longitudinal and transverse CV restitution. ASD = atrial septal defect; RA = right atrium; LA = left atrium; CV = CV.

For longitudinal conduction, local maximal CV was greater in both atria in the ASD group compared to the control group (RA: 0.61 ± 0.25 m/s vs. 0.39 ± 0.11 m/s; P = 0.016; LA: 0.65 ± 0.28 m/s vs. 0.47 ± 0.17 m/s; P = 0.046) (Figure 4A). There was no significant difference between right vs. left atrial local maximal CV in the ASD group (P = 0.66) or control group (P = 0.11).

For transverse conduction, local maximal CV was similar in both atria between the ASD group compared to the control group (RA: 0.31 ± 0.13 m/s vs. 0.27 ± 0.11 m/s, P = 0.41; LA: 0.31 ± 0.16 m/s vs. 0.23 ± 0.12 m/s, P = 0.11) (Figure 4B). There was also no significant difference in transverse CV between the right and left atria in either the ASD group (P = 0.95) or the control group (P = 0.34).

### Conduction velocity restitution

The maximal slope of CV restitution curves, indicating the rapidity with which conduction slows as a function of increasing activation rate, was calculated for both longitudinal and transverse local conduction.

For longitudinal conduction, the maximal slope of the CV restitution curves was greater in ASD patients in both atria (RA: 8.41 ± 4.2 ×10^−3^ ms^-2^ vs. 3.38 ± 2.5 ×10^−3^ ms^-2^, P < 0.01; LA: 9.71 ± 7.4 ×10^−3^ ms^-2^ vs. 4.77 ± 2.7 ×10^−3^ ms^-2^, P = 0.041) (Figure 4C). There was no significant difference in LA compared to RA maximal CV restitution curve slope in either the ASD group (P = 0.51) or the control group (P = 0.17).

For transverse conduction, there was no difference in the maximal slope of the CV restitution curves between ASD and control patients in either atrium (RA: 2.57 ± 1.7 ×10^−3^ ms^-2^ vs. 1.86 ± 1.1 ×10^−3^ ms^-2^, P = 0.24, LA: 3.19 ± 2.7 ×10^−3^ ms^-2^ vs. 1.86 ± 0.89 ×10^−3^ ms^-2^, P = 0.13). There was no significant difference in maximal CV restitution curves slope between the LA and RA in either the ASD group (P = 0.43) or the control group (P = 0.99) (Figure 4D).

### Effective refractory periods

**FIGURE 5:**
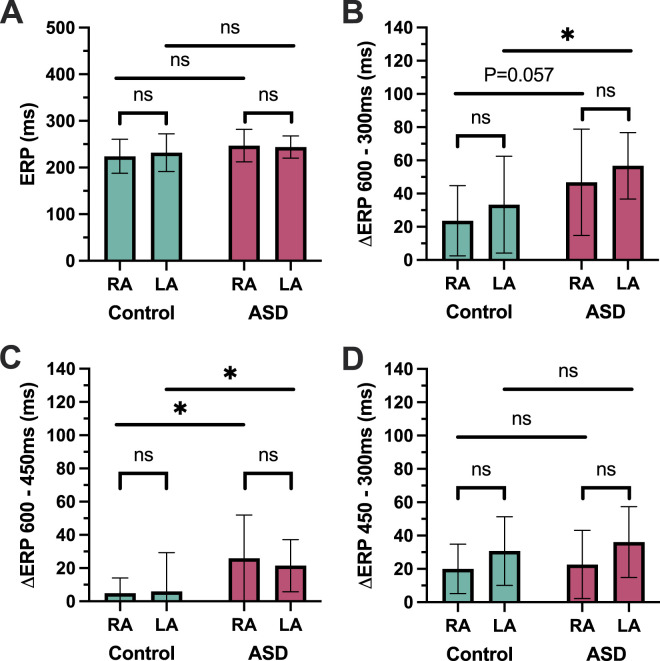
Repolarisation. **(A)** Right and left atrial effective refractory periods. **(B,C,D)** Change in refractory periods between pairs of basic cycle lengths. ASD = atrial septal defect; RA = right atrium; LA = left atrium; ERP = effective refractory period.

There was no statistically significant difference in effective refractory periods in either atrium between the ASD and control groups at any of the basic cycle lengths tested (Table 2). In both atria, ERP restitution slope was greater in ASD vs. control patients, largely driven by a greater decrease in ERP between 600 and 450 ms in both chambers (RA: 26.0 ± 26.0 ms vs. 5.0 ± 9.1 ms, P = 0.025; LA; 21.5 ± 16.7 ms vs. 6.0 ± 23.3 ms, P = 0.029) (Figure 5).

**TABLE 2:** Effective refractory periods.

	ASD	Control	P value
Right atrial ERP at 600 ms	247 ± 34.7	224 ± 36.5	0.071
Left atrial ERP at 600 ms	244 ± 23.9	232 ± 40.4	0.293
Right atrial ERP at 450 ms	220 ± 35	206 ± 21.1	0.200
Left atrial at 450 ms	222 ± 26.7	229 ± 33.5	0.511
Right atrial ERP at 300 ms	200 ± 26.6	193 ± 24.1	0.525
Left atrial at 300 ms	186 ± 13.3	197 ± 31.5	0.343

ASD, atrial septal defect; ERP, effective refractory period.

### Multivariable analysis

After controlling for age and co-morbidities through multivariable logistic regression, mean atrial voltage was independently associated with global CV in both atria of control patients (LA: 0.093 m/s decrease in CV for every 1 mV decrease in mean chamber voltage (P = 0.042); RA: 0.15 m/s decrease in CV for every 1 mV decrease in chamber voltage (P = 0.018)). However, mean atrial voltage was not an independent predictor of RA CV in ASD patients (LA: 0.11 m/s decrease in CV for every 1 mV decrease in mean chamber voltage (P < 0.01); RA 0.048 m/s decrease in CV for every 1 mV decrease in mean chamber voltage (P = 0.18)) (Table 3).

**TABLE 3:** Multivariable logistic regression.

	Right atrium			Left atrium		
	Beta	95% CI	P	Beta	95% CI	P
ASD group						
Age	−9.97 × 10^−4^	−3.37 × 10^−3^ – 1.37 × 10^3^	0.42	−1.22 × 10^−3^	−2.70 × 10^−3^ – 2.43 × 10^3^	0.12
Sex	−6.28 × 10^−3^	−0.0883 – 0.0760	0.88	−8.94 × 10^−3^	−0.057 – 0.0399	0.72
Hypertension	0.0279	−0.0537 – 0.110	0.51	−0.0334	−0.0770 – 0.0101	0.15
DM	−0.0692	−0.177 – 0.388	0.23	−0.0714	−0.127–−0.0101	0.024
CAD	0.210	0.0621–0.358	0.013	0.121	0.0450–0.196	<0.01
Mean voltage	0.0480	−0.0188 – 0.155	0.18	0.106	0.0463–0.165	<0.01
Control group						
Age	−3.59 × 10^−4^	−4.42 × 10^−3^ – 5.14 × 10^3^	0.89	−1.14 × 10^−3^	5.52 × 10^−3^ – 3.24 × 10^3^	0.62
Sex	3.12 × 10^−3^	−0.110 – 0.166	0.96	−0.0566	−0.167 – 0.0543	0.33
Hypertension	−0.0232	−0.137 – 0.0908	0.70	−0.170	−0.283–−0.0573	<0.01
DM	0.150	−0.110 – 0.409	0.28	0.268	2.72 × 10^−3^ – 0.534	0.065
CAD	−0.0420	−0.196 – 0.112	0.60	0.0380	−0.117 – 0.193	0.64
Stroke/TIA	−0.102	−0.314 – 0.109	0.36	0.0878	−0.0801 – 0.256	0.32
Mean voltage	0.152	0.0393–0.265	0.018	0.0926	0.0105–0.175	0.042

ASD, atrial septal defect; DM, diabetes mellitus; CAD, coronary artery disease; TIA, transient ischaemic attack.

## Discussion

This study characterises the electrophysiological properties of the right and left atria in patients with a secundum ASD. The main findings are.

1. Right atrial electroanatomic remodelling is present in ASD patients, characterised by increased RA low voltage areas, volume and surface area compared to control patients with already established paroxysmal AF.
2. Measurement of electrophysiological properties at fixed cycle lengths demonstrated no differences in either global or local transverse conduction in ASD patients compared to control patients.
3. Significant differences were observed in both CV and effective refractory period restitution properties in ASD compared to control patients.
4. In multivariable analysis, atrial structural remodelling (atrial voltage) independently correlated with CV in both atria in paroxysmal AF patients, but not in the RA of ASD patients.

These findings report, for the first time, the presence of reduced right atrial voltage in ASD patients and reveal significant differences in dynamic electrophysiological properties in ASD patients. In contrast to paroxysmal AF patients, atrial low voltage is not independently associated with CV in the remodelled RA of ASD patients. Alternative factors, for example cellular electrophysiological remodelling contingent upon right atrial stretch, may additionally be present to explain the observed differences in dynamic electrophysiological properties in ASD patients.

### Voltage mapping

Only one prior publication reports voltage mapping in ASD patients. In 2009, Roberts-Thompson et al. performed LA mapping on 11 ASD patients without a history of atrial arrhythmia and compared to 12 normal heart control patients, with mapping limited to the left atrium and involving <100 points per map (Roberts-Thomson et al., 2009). They described reduced mean LA voltage with a greater degree of low voltage areas in ASD patients. Our study extends these findings with bi-atrial evaluation using high-resolution electroanatomic mapping in the contemporary era.

While the relationship between LA voltage, AF severity and procedural outcome is well established, the interaction between RA voltage and AF is not clearly defined. A small number of studies delineating bi-atrial electrical change in specific disease states have reported on reduced right (and left) atrial voltage in patients with mitral stenosis, pulmonary and systemic hypertension, obstructive sleep apnoea and heart failure (John et al., 2008; Medi et al., 2012; Wang et al., 2014; Dimitri et al., 2012; Yamaguchi et al., 2022). In ASD patients post-closure, low voltage zones are strongly associated with atrial flutter and macro re-entrant atrial tachycardia circuits (Kondo et al., 2020; Nakagawa et al., 2001). Although these low voltage areas commonly represent the site of surgical incision, increased rates of focal atrial tachycardia are also seen in congenital heart disease patients post-surgical repair often in areas of atrial fibrosis or scarring distant to incisional sites (de Groot et al., 2010) with tissue disarray and consequent conduction heterogeneity as purported mechanisms.

While low bipolar voltage may not always correlate with atrial fibrosis defined by other means, the findings of this study largely reflect a prior study reporting on a significant right atrial fibrotic arrhythmia substrate in uncorrected ASD patients, as defined by cardiac MRI (O'Neill et al., 2022a).

In this study, comparison was made with a ‘positive’ control group consisting of patients with known paroxysmal AF in contrast to the studies mentioned above that employed ‘normal heart’ control groups without AF. As such changes of electrical remodelling associated with AF were expected in our control population and it is therefore notable that the extent of bi-atrial low voltage was greater in the ASD patients than the AF patients studied here, despite a low prevalence of atrial arrhythmia and a significantly lower age, potentially underscoring the tendency toward arrhythmia in this group.

### Repolarisation

Atrial repolarisation plays a key role in arrhythmogenesis with shorter refractory periods promoting re-entry through reduction in tissue wavelength. In AF patients, ERP tends to shorten with progression from paroxysmal to persistent AF (Teh et al., 2012; Uhm et al., 2013). In models of atrial stretch, however, conflicting results are seen. Shortened refractory periods in association with high atrial pressures have been demonstrated in animal models (Ravelli and Allessie, 1997) however in humans, longer refractory periods are described in subjects with increased atrial dimensions secondary to chronic ventricular pacing (Sparks et al., 1999). Similarly, in the two prior studies of atrial electrophysiology in uncorrected ASD patients without AF, similar or prolonged ERPs were noted in both atria compared to normal heart controls (Roberts-Thomson et al., 2009), Morton et al. (2003) as described in mitral stenosis and atrial dilatation (John et al., 2008). Tissue fibrosis and associated disruption of CV may have a large impact on AF vulnerability in these patients, a theory supported by a canine study in which the role of regional conduction slowing in the development of AF was highlighted, despite the presence of unchanged or prolonged ERPs (Li et al., 1999).

The present study identifies a non-significant trend towards longer refractory periods at 600 ms in the ASD group, similar to that described by Morten et al., in 2003 (Morton et al., 2003), but is the first to describe ERP restitution properties in uncorrected ASD patients. Action potential duration (APD) restitution has been extensively studied in animal and simulation studies and the slope of the restitution curve identified as crucial to the stability of re-entrant circuits (Courtemanche, 1996; Frame and Simson, 1988) with steeper curves associated with spiral wave break-up and fibrillatory activity (Qu et al., 1999). ERP as a surrogate for APD has been validated in an atrial modelling study which identified the steepness of the ERP restitution curve as a reliable predictor of spiral wave break up (Xie et al., 2002). Steeper APD restitution curves have also been previously documented in both paroxysmal and ‘chronic’ AF patients compared to controls (Kim et al., 2002), and in the ASD patients studied here steeper ERP restitution than in patients with established AF may indicate cellular remodelling supporting arrhythmia.

### Conduction velocity

Changes in tissue conductivity and increased vulnerability to AF induction seen in the presence of conduction slowing and block are well described (Teh et al., 2012; Zheng et al., 2017). In uncorrected ASD patients, conduction times were significantly greater in both atria in ASD patients than in normal heart controls (Roberts-Thomson et al., 2009; Morton et al., 2003). In this study we used a recently described technique for measuring dynamic local CV properties in the atria (Gharaviri et al., 2022). The S1S2 stimulation protocol provides information on maximum CV and the slope of the CV restitution curve in multiple directions. Contrary to prior reports we noted greater maximum local CV in ASD vs. control patients. We also describe significantly steeper CV restitution curves in the ASD cohort in both chambers. In a clinical study of AF mechanisms, both broad and steep patterns of CV restitution were seen in AF patients in contrast to flat restitution slopes in non-AF controls (Lalani et al., 2012). Steeper restitution curves may allow for re-entry to be perpetuated, with flattening of the CV restitution curve noted to be protective against spiral wave break up and fibrillatory activity (Qu et al., 1999).

### Clinical significance

The management of atrial arrhythmias in patients with ASD is challenging. Closure alone does not appear to reduce the overall prevalence of arrhythmias, (O'Neill et al., 2020) whilst isoprenaline infusion does not reveal the predilection for right-sided ectopy which is evident during ambulatory monitoring in patients with ASD (O'Neill et al., 2022b). Given both this predilection for right-sided ectopy, and the degree of bi-atrial remodelling observed in ASD patients in this study, catheter ablation strategies routinely targeting the RA may be of added value in the ASD population. Indeed, in surgical ASD patients undergoing closure, right plus left atrial MAZE confers the greatest benefit in patients with AF, (Im et al., 2013) again highlighting the bi-atrial nature of the arrhythmia substrate. With RA re-entrant circuits and focal activations potentially linked to zones of low voltage in the ASD cohort, (Uhm et al., 2013) individually tailored substrate driven ablation may improve arrhythmia outcomes. To determine optimum therapeutic strategies in this cohort, a randomised controlled trial evaluating bi-atrial ablation strategies in ASD patients with arrhythmia is warranted.

## Limitations

The sample size in this study was limited to 22 ASD patients, and the ASD group was heterogenous with respect to the presence or absence of atrial arrythmias. The number of ASD patients with documented atrial arrhythmia was small, limiting the ability to draw conclusions on factors associated with arrhythmogenesis in the population studied. Furthermore, age and sex differences between ASD and control group may limit interpretability of the findings. Nevertheless, the data presented represents the largest cohort of uncorrected ASD patients undergoing invasive electrophysiological assessment, and the only simultaneous bi-atrial assessment of its nature in these patients to date. Due to intra-procedural time limitations, atrial ERP and local CV were measured at one site only per chamber, which may not fully reflect the electrophysiological changes present throughout the atria.

## Conclusion

This is the first study to describe the electrophysiological properties of the right atrium in ASD patients using electroanatomic mapping. The findings demonstrate the predominance of right atrial low voltage in ASD patients. Steeper ERP and CV restitution slopes are also seen in ASD patients compared to control paroxysmal AF patients. Further work is needed to evaluate the effects and timing of ASD closure on arrhythmogenesis and the role of right atrial ablation to improve arrhythmia outcomes in ASD patients.

## Clinical perspectives

### Clinical competencies

Competencies in medical knowledge; This work is the first to comprehensively describe bi-atrial electrophysiological properties using electroanatomic mapping in patients with atrial septal defects.

Competencies in patient care; The predominance of right atrial remodelling assessed by invasive voltage mapping in the ASD group may imply a future role for right atrial ablation in patients undergoing catheter ablation for atrial arrhythmias.

### Translational outlook

This study suggests that key differences are present in bi-atrial electrophysiology between ASD patients and normal heart AF control patients. As such optimum ablation strategies in the ASD population cannot be implied from the general AF population. The findings of this study may prove useful in informing further clinical studies of ablation strategies in ASD patients with atrial arrhythmia.

## Data Availability

The datasets presented in this article are not readily available because they contain human clinical data collected under an ethically approved research protocol, and access is subject to institutional and ethical approvals and appropriate data use agreements or sponsorship arrangements. Requests to access the datasets should be directed to the corresponding author.
